# Measuring internal inequality in capsule networks for supervised anomaly detection

**DOI:** 10.1038/s41598-022-17734-7

**Published:** 2022-08-09

**Authors:** Bogdan Kirillov, Maxim Panov

**Affiliations:** 1 Skolkovo Institute of Science and Technology, Moscow, Russia; 2 Center for Precision Genome Editing and Genetic Technologies for Biomedicine, Institute of Gene Biology, Russian Academy of Sciences, Moscow, Russia; 3 Technology Innovation Institute, Abu Dhabi, United Arab Emirates

**Keywords:** Applied mathematics, Computer science

## Abstract

In this paper we explore the use of income inequality metrics such as Gini or Palma coefficients as a tool to identify anomalies via capsule networks. We demonstrate how the interplay between primary and class capsules gives rise to differences in behavior regarding anomalous and normal input which can be exploited to detect anomalies. Our setup for anomaly detection requires supervision in a form of known outliers. We derive several criteria for capsule networks and apply them to a number of Computer Vision benchmark datasets (MNIST, Fashion-MNIST, Kuzushiji-MNIST and CIFAR10), as well as to the dataset of skin lesion images (HAM10000) and the dataset of CRISPR-Cas9 off-target pairs. The proposed methods outperform the competitors in the majority of considered cases.

## Introduction

The problem of anticipating rare events is of high interest to the modern technological society. A lot of problems people face, like bank fraud^1^, structural defects in materials^2^, early development of diseases^3^, and manipulation of public opinion in social networks^4^, boil down to knowing what a typical behavior for a system is and what is not.

Anomaly detection is the process of examining data to determine where the aberrations lie. Usually, this involves analyzing how well the parts of the system are performing to understand what the normal behavior consists of. Sometimes there is also some degree of knowledge about abnormal behavior. In this paper, we use the common notion of anomaly in machine learning—an instance of data that is rare and deviates a lot from other, more prevalent ones. Anomaly detection is essential for analysis of almost any complex data. In bioinformatics, one can consider prediction of protein–protein interactions and CRISPR off-target cleavage prediction. In computer vision there are various cases of defect detection. All these tasks require a deep neural network-based solution due to the data complexity.

Anomaly detection then can be supervised or unsupervised^5^ depending on whether the examples of atypical behavior are available. Each kind has its benefits and limitations: supervised anomaly detection methods tend to be more accurate with the known anomalies than the unsupervised ones, but also tend to miss the anomalies never observed before^6^.

Our paper is concerned with supervised anomaly detection, which deals with the classification problems of a very abundant normal class and a scarce anomalous class. It is a case of highly imbalanced binary classification. We focus on the problems with relatively complex data such as images or DNA sequences, which are best solved with Deep Learning methods. Given that the anomalous class is usually infrequent (1–0.01%, mere hundreds of examples), commonly used deep learning methods tend to perform poorly. Supervised anomaly detection via deep neural networks usually employs carefully crafted augmentation^7^, complex architectures^8^, GAN-based generation^9^, and other tricks aimed to expand the number of anomalous examples.

In this work, we present a different approach, based on capsule networks with dynamic routing^10^. A capsule network consists of grouped neurons that output vectors encoding parameters of an object or a part of an object. The key difference between Capsule and Convolutional Neural Networks is the output: while Convolutional Networks output a vector of $N$ class probabilities (where $N$ is the number of classes), capsule networks output a matrix that consists of $N$ vectors. These vectors are called *capsules* and encode the learned representation of an object given it belongs to a corresponding class. The class probabilities are computed by taking the vector norms. Low-level primary capsules that represent parts of an object feed their output to class capsules that represent the object as a whole. Parts from an object of a rare class are rarely present in an object of a more common class. If the network detects parts that do not fit into a common class, then the low-level primary capsules that correspond to these parts are triggered and therefore contribute to prediction of the object being an anomaly. A method to detect anomalies with Capsule Networks would benefit from exploiting this “part-whole” relationship expressed in dynamics of primary capsules voting for a class.

Previous works^11,12^ on supervised anomaly detection with capsule networks use the reconstruction ability and class probabilities to separate outliers from inliers, while the methods proposed in this work are based on the evaluation of unequal response of the routing mechanism to normal and aberrant inputs. Class probability is given by the computation of class capsule output via routing. Routing by agreement has an intrinsic property of polarization^13^—convergence on a single route from primary to class capsules. This property gives rise to inequality between a well-predicted and poorly predicted class in case of class imbalance. We can measure such discrepancy using economic inequality metrics, such as Gini^14^ and Palma^15^ coefficients. Our main contributions can be summarized as follows:

1. We propose a new approach for supervised anomaly detection using capsule networks;
2. We suggest a new application of economic inequality metrics to machine learning which also allows investigating internal mechanisms of capsule networks;
3. We perform a comprehensive review and comparison of different capsule network-based anomaly detection methods on standard benchmarks and real-world data, which confirms state-of-the-art performance of the proposed methods.

## Materials and methods

### Capsule networks as an ensemble of weak learners

**Figure 1:**
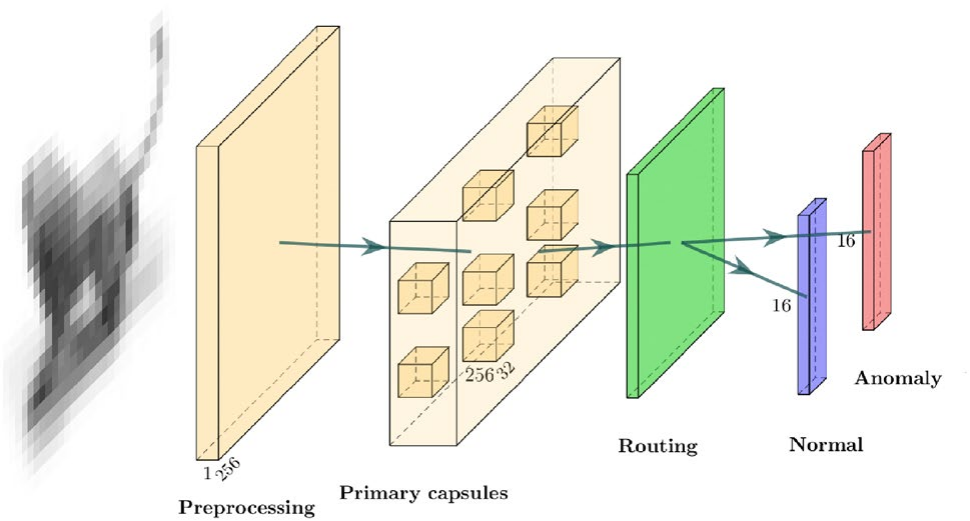
Capsule Networks architecture. Primary capsules are formed out of a convolutional layer set. Those layers have the same input and their output is combined for routing. The decoder is not shown. Architecture for MNIST-like experiments is shown here and the changes we made for different cases are described in the respective sections.

The main difference of Capsule Networks from conventional architectures is the output. The output is not probabilities of classes, but rather vectors with information on learned features. This is achieved through combining the output of different small models and summarizing their decisions. There are two types of capsules, primary and secondary, so Capsule Networks are inherently two-layered. Class capsules perform prediction based on information from all primary capsules combined. The output of primary capsule acts as an asset for a class capsule $N$ in a way that it enlarges or shrinks its output vector, giving a large or small probability of an example belonging to class $N$. Different primary capsules are of different value for class capsule $N$ and the vectors of class capsules act as a kind of summary statistics over the distribution of primary capsule contributions. We hypothesize that the distribution of the “values” in case of a normal example will be different than in case of an anomalous one, so we can detect anomalies based on that difference. The architecture of Capsule Networks proposed by Sabour et al.^10^ is shown in Fig. 1.

In case of image classification, capsule networks process the input image in three steps: preprocessing, routing and reconstruction. Preprocessing in our setting consists of a 2D convolutional layer followed by an activation function (ReLU or ELU in our experiments). It is followed by a number of 2D convolutional layers of $N_{f}$ filters, which produces the output of size ($N_{f}$, output width $w$ and output height $h$). The outputs of every convolutional layer are joined together and flattened out to form a tensor of size (number of primary capsules $N_{P} = N_{f} \times w \times h$, dimension of primary capsule output $O_{P}$). Then the squash activation function is applied element-wise separately for each element in batch and for each 2D convolutional layer:

1 $$\begin{aligned} S(x) = \frac{\left\Vert x\right\Vert ^{2}}{1 + \left\Vert x\right\Vert ^{2}} \frac{x}{\left\Vert x\right\Vert }, \end{aligned}$$

where *x* is the input to the squash activation, e.g. output of the preprocessing stage. The outputs of primary capsules are then fed into $N_{S}$ secondary or class capsules (in our setting $N_{S} = 2$) and we enter the stage of routing. The routing is an iterative algorithm and the following steps describe a single iteration. Usually the routing is repeated for 3 iterations.

During the routing we first compute the coupling coefficients—tensor $c$ of shape ($N_{P}, N_{S}, O_{S}$). For each secondary capsule $j$ we compute the prior probability of coupling for all primary capsules and save it into the coupling coefficient table. We will use the following key constructions below:

1. $c_{ij}$—a slice of tensor $c$ that corresponds to primary capsule $i$ and secondary capsule $j$;
2. $W_{ij}$—the corresponding slice of the weight tensor;
3. $u_{i}$—an output of primary capsule $i$.

Then for each secondary capsule $j$ we compute *unsquashed* secondary capsule output using the product of the weight tensor slice that corresponds to the primary capsule $i$ and secondary capsule $j$ and the outputs of the primary capsules $u_{i}$:

$$\begin{aligned} s_{j} = \sum _{i = 1}^{N_{P}} c_{ij} \odot (W_{ij} u_{i}), \end{aligned}$$

where $\odot$ denotes elementwise multiplication.

The outputs of secondary capsules are computed as in case of primary ones, by the squashing function [see Eq. (1)] for every secondary capsule $j$:

$$\begin{aligned} v_{j} = S(s_{j}), \; j = 1, \dots , N_{S}. \end{aligned}$$

The “agreement” is computed by scalar multiplication of outputs from secondary capsule $j$ with the product of weights and primary capsule outputs that correspond to primary capsule $i$ and the secondary capsule, and at the end of the routing iteration the routing table is updated.

To get the probabilities of classes for an example, we should compute the Euclidean norm of the vectors we get from secondary capsules:

$$\begin{aligned} P(Y=C_{j}) = \frac{\exp \left( \left\Vert v_{j}\right\Vert \right) }{\sum _{k=1}^{N_{S}} \exp \left( \left\Vert v_{k}\right\Vert \right) }, \end{aligned}$$

where $C_{j}$ is the class label that corresponds to $j$-th class capsule.

#### Loss function and regularization

We can train the network with any kind of classification objective but following Sabour et al.^10^ we use margin loss:

$$\begin{aligned} L_{k} = T_{k} ReLU\left( m^{+} - \hat{Y_{k}}\right) ^{2} + \lambda \left( 1 - T_{k}\right) ReLU\left( \hat{Y_{k}} - m^{-}\right) ^{2}, \end{aligned}$$

where $k$ is the number of output capsule, $T_{k} = 1$ if the k-th capsule denotes the class that corresponds to the real label and 0 otherwise, $m^{+}$, $m^{-}$ and $\lambda$ are hyperparameters, $\hat{Y_{k}}$ is the norm of the $k$-th capsule output vector. Full description of all hyperparameters used can be found in the Supplementary Note.

As a regularization, like in the original paper^10^, the additional reconstruction subnetwork $R$ is used:

$$\begin{aligned} \hat{X} = R(v_{j}). \end{aligned}$$

So the total loss we optimize is:

$$\begin{aligned} L_{total} = \sum _{k = 1}^{N} L_{k} + \alpha MSE(X, \hat{X}), \end{aligned}$$

where $\alpha > 0$ is a hyperparameter chosen to balance the involvement of reconstruction loss. Most of the learning happens when the weights of the class capsule layer are adjusted via gradient descent during the backward pass. The adjustment is based on the output of the layer during the forward pass. The forward pass is computed iteratively, according to the equations above. The usual way to train capsule networks is not easy: one needs to use a non-standard loss and an additional decoder for regularization, but the most interesting computations happen within the coupling coefficients.

### Main idea

Both previous studies^11,12^ base their work on the differences between estimated class probabilities and reconstruction subnetwork. We do not use reconstructions in our method while it could indeed help in a real-life application. Instead, we focus on the estimation of class probabilities. In capsule network setting, the probabilities are formed by softmax of capsule output vector norms. Output vectors provide information beyond class probability, according to the original Capsule Networks paper^10^, they capture interpretable properties like thickness of stroke, localization and shape. This information gets lost when we compute the norm.

To gather as much information as possible, we dive deeper into the routing mechanics. Coupling coefficients $c$, computed from the routing table, contain all the information about the way primary and class capsules would route for a given example. We base our research on the assumption that the couplings on normal and anomalous capsule show different results when encountered with an abundant class of examples and a rare one.

**Figure 2:**
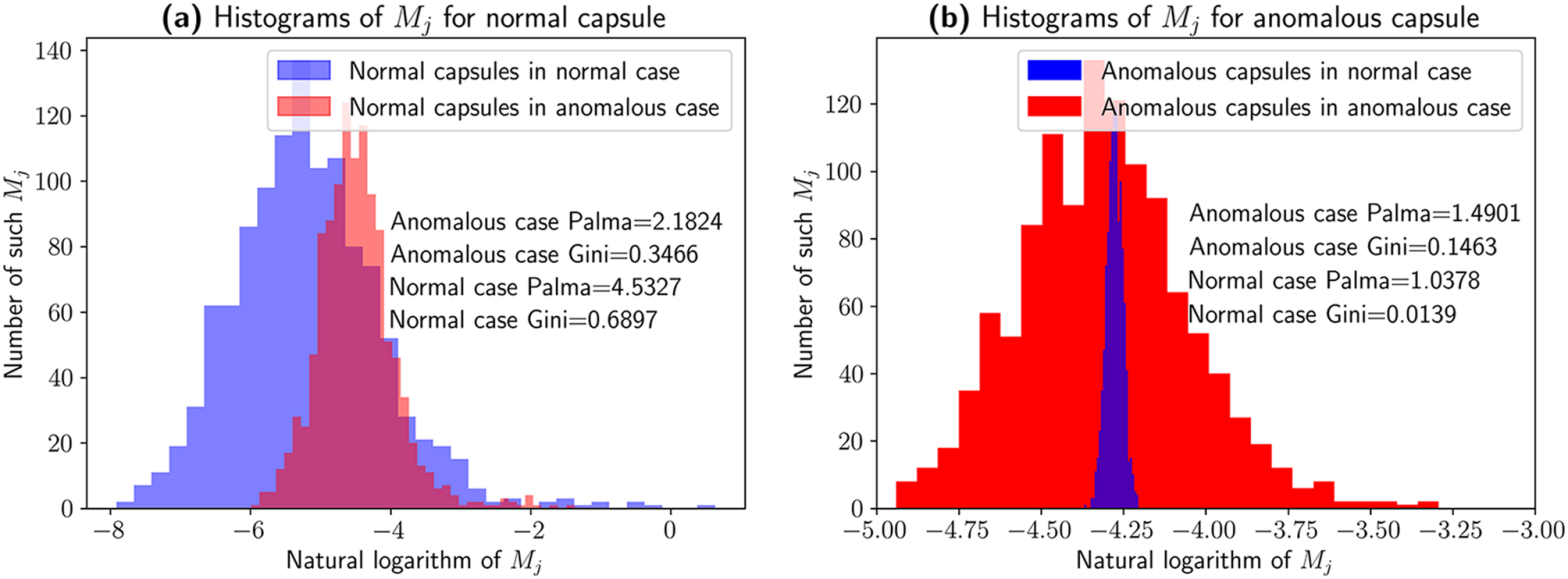
An example of coupling coefficients distributions $M_{j}$ for the data from KuzushijiMNIST. The histograms of $M_{j}$ [Eq. (2)] for normal and anomalous capsules in normal and anomalous cases are shown with specified Gini and Palma coefficients. Note the difference between the distributions for normal (**a**) and anomalous (**b**) capsules in the normal case and the anomalous one. Normal capsule only mildly captures the difference between the samples. We use the couplings of anomalous capsule to compute Gini and Palma coefficients to capture this more pronounced difference for anomalous capsule. Note that on the plot, the distribution for the logarithm of the summed couplings are shown, but the values of Gini and Palma coefficients given are computed on the summed couplings without the logarithm.

To do the classification, we ideally need a summary statistics for the couplings. Let $c_{j}$ be a part of coupling table $c$ that corresponds to $j$-th secondary capsule. It is a tensor of size $(N_{P}, O_{S})$ where $N_{P}$ is the number of primary capsules, $O_{S}$ is the dimension of class capsule output vector. For each secondary capsule $j$ we first sum the respective couplings along the $O_{S}$ axis:

2 $$\begin{aligned} M_{j} = \sum _{l = 1}^{O_{S}} c_{j}^{l}, \end{aligned}$$

where $c_{j}^{l}$ is a vector of dimension $N_{P}$ obtained as a slice of tensor $c$ for secondary capsule $j$ and its output dimension $l \in \overline{1, N_{S}}$. The distribution of $M_{j}$ for different cases is shown on the Fig. 2 below. This vector can be interpreted as a vector of total contributions of primary capsules to the $j$-th secondary capsule results. Those contributions due to polarization property of capsule networks would be highly inequal in case the network is well-trained. Figure 2 clearly shows the significant change in the distribution for the anomalous capsule that can be captured by inequality measures discussed below.

To evaluate an internal inequality in capsule networks, we require a short detour to econometrics. Income inequality in economics is measured by a number of statistical criteria^15^. Most popular, the ones we use here, are Gini^14^ and Palma^16^ coefficients. Gini coefficient is

3 $$\begin{aligned} Gini(Z) = \frac{\sum _{i = 1}^{n} (2i - n - 1) Z_{i}}{n \sum _{i = 1}^{n} Z_{i}}, \end{aligned}$$

where $n$ is the sample size, $i$ is the number of example in the sample and $z_{i}$ is the value. Gini coefficient ranges from 0 to 1 and the value for the most inequal case (only one non-zero example) is 1.

More recently, Palma^16^ coefficient started to displace Gini as a go-to measure of income inequality:

4 $$\begin{aligned} Palma(Z) = \frac{Q_{90}(Z)}{Q_{40}(Z)}, \end{aligned}$$

where $Q_{90}$ and $Q_{40}$ are the 90-th and 40-th percentiles respectively. Key assumption behind Palma coefficient is that the tails of income distribution contribute to inequality the most and the middle ground remains relatively stable over time. This assumption makes Palma coefficient work rather well in case of yearly assession of economic inequality of countries. We hypothesise that Palma coefficient would work better than Gini because the assumption holds in our case due to polarization.

Now we are equipped to apply income inequality metrics to Capusle Networks. The first proposed criterion is based on Gini coefficient, see Eq. (3):

$$\begin{aligned} Gini(M_{j}) = \frac{\sum _{k=1}^{N_{P}}(2k - N_{P} - 1) M_{jk}}{N_{P} \sum _{k = 1}^{N_{P}} M_{jk}}. \end{aligned}$$

The second criterion is based on Palma coefficient and is computed according to Eq. (4):

$$\begin{aligned} Palma(M_{j}) = \frac{Q_{90}(M_{j})}{Q_{40}(M_{j})}. \end{aligned}$$

Coming back to the example from Fig. 2, we clearly see that both Gini and Palma coefficients allow to capture the difference in distribution for anomalous capsule.

We use both of these criteria as a measure of data point “outlierness” and compute the AUC directly. It is possible to use Logistic Regression or any other classification model (SVM, XGBoost, Random Forest, ...) based on the value of Gini, Palma or both and also consider adding other features derived from the data or reconstruction properties, but we defer it to future work.

### Data

#### MNIST-like benchmarks

The previous studies^11,12^ are conceptually similar but they offer different ways to measure the performance of the anomaly detection metrics. We first considered an experiment inspired by the work of Piciarelli et al.^12^ which is organized following the *model generation procedure for Diverse Outlier setup*:

1. Extract all examples of class *i* from the training set with *N* classes, and assign the label $l=0$;
2. Randomly extract *A* examples of any other class and assign the label $l=1$;
3. Train a model to classify the data into two classes.

We apply this procedure to all classes in the datasets and to the fractions of 10%, 1% and 0.1% so we get $4 \times 3 \times 10 = 120$ models to test our results on. This procedure gives us the coherent normal subset and a diverse subset of outliers.

Approach based on the work of Li et al.^11^ is the reverse one in nature—we use a single class for our anomaly label and all other classes we consider normal, so our normal set is diverse and anomalous set is coherent. We train the models following a *model generation procedure for Diverse Inlier setup*. This gives us another 120 models to test:

1. Extract all examples of class *i* from the training set with *N* classes and assign the label $l=1$;
2. Randomly extract *A* examples of all other classes and assign the label $l=0$;
3. Train a model to classify the data into two classes.

We use MNIST^17^, FashionMNIST^18^, KuzushijiMNIST^19^ and CIFAR10^20^ with the diverse outlier and diverse inlier setups to make a comparison of the proposed methods, the previous studies^11,12^ and the baselines. Each dataset except CIFAR10 has 60,000 single-channel images of (28,28) size that are separated into 10 classes. CIFAR10 has 60,000 images with 3 channels and (32,32) size, also separated into 10 classes.

#### Malignant skin lesion classification

The HAM10000^21^ dataset contains high-quality photos of 7 skin lesion types three of whom are malignant. The dataset contents are shown on Table 1.

**Table 1:** Detailed information on classes for HAM10000 dataset.

Skin lesion type	# Images	Is malignant?
Melanoma	1113	Yes
Basal cell carcinoma	514	Yes
Dermatofibroma	115	No
Melanocytic nevus	6705	No
Vascular lesion	142	No
Benign keratosis-like	1099	No
Intraepithelial carcinoma	327	Yes

Inspired by the anomaly-based cancer detection pipeline^3^, we consider malignant skin lesions anomalies, aberrations of the correct skin cell life-cycle and while benign skin lesions are also a kind of an aberration, we consider them a base for the normal classes in our experiments. From this dataset we derive four experiments:

1. Diverse normal set, diverse anomalous set: all malignant types as an anomaly set, all benign types as a normal set;
2. Diverse normal set, homogeneous anomalous set: melanoma (the most common skin cancer) images as an anomaly set, all benign types as a normal set;
3. Homogeneous normal set, diverse anomalous set: all malignant types as an anomaly set, melanocytic nevus (the most common benign lesion, a birthmark) images as a normal set;
4. Homogeneous normal set, homogeneous anomalous set: melanoma vs melanocytic nevus.

#### CRISPR-Cas9 off-target detection

Off-target cleavage in CRISPR/Cas9-based gene editing can lead to various unforeseen consequences. For a design of gene editing experiment it is of high importance to select such gRNAs that minimize the probability of Cas9 performing doublestrand cleavage in a wrong place (off-target effect). To do so using Machine Learning, a dataset of gRNA-target pairs is used. In this setting we classify the pairs in two classes: no cleavage(0) and an off-target(1).

The dataset of CRISPR-Cas9 off-targets taken from the work of Peng et al.^22^ consists of 215 low-throughput off-target pairs, 527 high-throughput off-target pairs and a negative subset of 408,260 pairs. The low and high throughput pairs are labelled 1 and the negative subset thus 0. Each pair is two strings of “A”, “T”, “G” and “C” symbols. We convert pairs into images using the following preprocessing routine:

1. One-hot encode the target string (which is 23 nucleotides long) to get $I_{1}$;
2. One-hot encode the gRNA string (which is also 23 nucleotides long) to get $I_{1}$;
3. Join the encodings to get a tensor of size ($2,4,23$).

We do not use the pairs that have more than 6 mismatches (since the off-target cleavage is considered to be impossible in that case) so the final dataset consists of 615 anomalous pairs (off-targets) and 26,038 normal pairs from negative subset.

## Related work

Within the supervised framework, a model for anomaly detection is trained to discriminate normal examples from the anomalous ones. It has a certain advantage in the discrimination ability over the model within the unsupervised framework and in cases where the anomalies are rather homogeneous, it usually performs the best. One of the basic methods in supervised anomaly detection using deep learning is Negative Learning (NL^23^)—to distinguish between the outliers and the normal data points, one uses the reconstruction error of autoencoder that is trained to reconstruct normal data points perfectly while failing to reconstruct the outliers. Negative learning-based anomaly detection does suffer from inability to reconstruct normal data points though. To overcome this issue, the work of Yamanaka et al.^6^ introduces the Autoencoding Binary Classifiers (ABC) which extend the negative learning approach by providing lower and upper boundaries on the loss function with respect to the reconstruction errors. NL and ABC will be used in our work as the baselines.

The main idea of negative learning^23^ is to permanently damage the reconstructive ability of an autoencoder by forcing it to maximize the reconstruction error on anomalous samples while minimizing it on normal ones. As an autoencoding model, the work of Munawar et al.^23^ uses Restricted Boltzmann Machine with a visible layer and a hidden layer. The network is trained using single-step contrastive divergence^24^:

$$\begin{aligned} \delta w = \sigma \left[ {{\,\mathrm{\mathbb {E}}\,}}\left[ (vh)_{original} - (vh)_{reconstructed}\right] \right] , \end{aligned}$$

where *v* is the visible layer, *h* is the hidden layer, $\sigma$ is the sign and $\delta w$ is the gradient of weights. For positive learning stage, $\sigma = 1$, for negative $-\sigma = -1$. During one training pass, the positive learning is done first, on all positive examples, then the negative learning is done on all negative examples. Autoencoding Binary Classifier uses the following loss, constrained in case of anomalous input:

$$\begin{aligned} L_{ABC}(X, Y) = Y MSE(X, \hat{X}) - (1 - Y) \log \Bigl (1 - e^{-MSE(X, \hat{X})}\Bigr ). \end{aligned}$$

The logarithm term caps the loss for the $Y \ge 1$. Additionally, the ABC paper^6^ uses multilayer perceptrons instead of RBMs for architecture and gradient descent instead of CD for training algorithm.

Capsule networks were already used for anomaly detection in the works of Piciarelli et al.^12^ and Li et al.^11^. The first paper considers supervised anomaly detection setup while the second one proceeds with an unsupervised formulation. They propose anomaly and normality metrics respectively, that are based on usage of regularizing decoder and the difference between the estimated probabilities of normal and anomalous class. In our paper we show that those metrics are a direct consequence of internal inequality of “assets” in Capsule Network representations. The probabilities and reconstruction errors are on the end of the pipeline and a lot of information that help distinguish the anomalies from normal data is lost during the process of their computation.

We compare our work with both previous studies^11,12^ and a few baselines (NL and ABC). Our working hypothesis implies that the information ignored when the probabilities are computed helps detect anomalies better. We also compute all available anomaly metrics and show that our work provides the best results in most cases. The work of Piciarelli et al.^12^ proposes the following *anomaly measure*:

5 $$\begin{aligned} A\left( X,\hat{X}, \hat{Y}_{normal}, \hat{Y}_{anomaly}\right) = \hat{Y}_{normal} - \hat{Y}_{anomaly} + MSE(X, \hat{X}), \end{aligned}$$

where $X$ is the input image, $\hat{X}$ is its reconstruction, $\hat{Y}_{n}, n\in \{normal,anomaly\}$—the norm of capsule output vectors. It is based on the observation that for an anomaly the difference between probabilities for each class tends to be less drastic than for a normal example. Additionally, this paper^12^ proposes filtering the anomaly class from the input to the reconstruction so the reconstruction network is trained to reconstruct only normal images. We include this feature to every experiment with Capsule Network.

The work of Li et al.^11^ provides two metrics. The first one, given by Eq. (6), is the largest probability of a class:

6 $$\begin{aligned} N_{PP}(\hat{Y}) = \max _{i = 1, \dots , N_{S}} \hat{Y}_{i}, \end{aligned}$$

where $\hat{Y}$ is the vector of norms of the capsule output vectors and $N_{S}$ is the number of output capsules.

Equation (6) is introduced under the assumption that for a normal example there would be only one capsule with the norm close to 1 and for an outlier both of capsules would be close to each other. Hence, for normal images the results of Eq. (6) would be close to 1, but for an outlier they would be close to 0.5. The authors of the previous work^11^ normalize MSE by the euclidean norm of inputs in Eq. (7), because the MSE is dependent on the number of non-background pixel in input and reconstruction and the authors tried to invent a metric that is unaffected by this issue.

7 $$\begin{aligned} N_{RE}(X, \hat{X}) = \frac{MSE(X, \hat{X})}{\sqrt{\sum _{i = 1}^{n} X_{i}^{2}}}, \end{aligned}$$

where $X$ is an input image and $\hat{X}$ is the reconstruction computed by the reconstruction subnetwork.

We compare the proposed methods with a selected set of previous works^6,23^ because those works provide clear, simple and accurate approaches that are similar enough to ours so the design of a comparison study is pretty straight-forward.

## Results

### MNIST-like benchmarks

We measure the performance using the AUROC metrics. For each dataset and outlier fraction we compute AUROCs for all classes (10 values), then we report average AUROC and the standard deviation. We denote performance of the capsule network without any additional metrics as “Plain”, non-capsule baselines as NL and ABC, anomaly score as A, normality scores as $N_{pp}$ and $N_{re}$. The results for diverse outliers are shown in Table 2.

**Table 2:** AUROCs for diverse outlier setup (fractions 0.1%, 1% and 10%).

	0.1%, mean ± std	1%, mean ± std	10%, mean ± std	Dataset
Palma	**0.6488** ± 0.0598	0.7213 ± 0.0644	**0.8313** ± 0.0473	CIFAR10
Gini	**0.6494** ± 0.0604	**0.7217** ± 0.0646	**0.8322** ± 0.0471	
Plain	0.5000 ± 0.0000	0.5003 ± 0.0009	0.6372 ± 0.0635	
A	0.5813 ± 0.1159	0.5856 ± 0.1207	0.6084 ± 0.0985	
$N_{pp}$	0.6487 ± 0.0755	**0.7369** ± 0.0644	0.7915 ± 0.0581	
$N_{re}$	0.5851 ± 0.1085	0.5908 ± 0.1198	0.6159 ± 0.0944	
ABC	0.6407 ± 0.0714	0.6426 ± 0.0772	0.6489 ± 0.0707	
NL	0.5000 ± 0.0811	0.5000 ± 0.0811	0.5000 ± 0.0812	
Palma	0.8387 ± 0.0876	0.9795 ± 0.0123	0.9805 ± 0.0221	MNIST
Gini	0.8366 ± 0.0883	**0.9819** ± 0.0131	**0.9984** ± 0.0007	
Plain	0.5767 ± 0.0660	0.8633 ± 0.0584	0.9821 ± 0.0060	
A	**0.9253** ± 0.0522	0.9545 ± 0.0313	0.9541 ± 0.0392	
$N_{pp}$	0.9144 ± 0.0644	0.9528 ± 0.0297	0.7479 ± 0.0929	
$N_{re}$	**0.9618** ± 0.0235	**0.9806** ± 0.0130	**0.9896** ± 0.0087	
ABC	0.8169 ± 0.1073	0.8102 ± 0.1051	0.8293 ± 0.1063	
NL	0.4943 ± 0.1723	0.4947 ± 0.1714	0.4942 ± 0.1716	
Palma	0.7458 ± 0.0489	**0.9109** ± 0.0299	**0.9679** ± 0.0217	KMNIST
Gini	0.7446 ± 0.0494	0.9096 ± 0.0301	**0.9757** ± 0.0154	
Plain	0.5048 ± 0.0098	0.6642 ± 0.0692	0.9071 ± 0.0362	
A	0.7585 ± 0.0764	0.7881 ± 0.0572	0.7870 ± 0.0764	
$N_{pp}$	**0.8417** ± 0.0486	**0.9316** ± 0.0247	0.7931 ± 0.0548	
$N_{re}$	**0.8645** ± 0.0547	0.8923 ± 0.0461	0.9066 ± 0.0391	
ABC	0.6025 ± 0.1122	0.6049 ± 0.1127	0.6180 ± 0.1215	
NL	0.5000 ± 0.1078	0.5000 ± 0.1072	0.5000 ± 0.1067	
Palma	0.8388 ± 0.1134	**0.9390** ± 0.0467	**0.9602** ± 0.0340	FMNIST
Gini	0.8419 ± 0.1119	**0.9392** ± 0.0468	**0.9784** ± 0.0271	
Plain	0.5759 ± 0.1172	0.8027 ± 0.0797	0.9210 ± 0.0554	
A	**0.8914** ± 0.0609	0.9090 ± 0.0570	0.9087 ± 0.0820	
$N_{pp}$	0.8792 ± 0.0567	0.8834 ± 0.0836	0.7453 ± 0.1117	
$N_{re}$	**0.9153** ± 0.0666	0.9195 ± 0.0715	0.9080 ± 0.0875	
ABC	0.7921 ± 0.1595	0.7921 ± 0.1588	0.7933 ± 0.1611	
NL	0.5000 ± 0.2215	0.5000 ± 0.2218	0.5000 ± 0.2207	

The best and the second-best results are in [bold].

The proposed methods, either Palma or Gini outperforms other metrics and baselines in 1% and 10% cases for diverse outliers for both AUROC and average precision (shown in Supplementary Table 1). For CIFAR10, Palma and Gini also perform the best in 0.1% case. This is probably due to loss of information after computing the norms according to Eqs. (5) and (6). For 1% KMNIST and 1% CIFAR10 case, Palma and Gini respectively come second to $N_{pp}$. In MNIST and FMNIST 0.1% though (in AUROC, and for KuzijishiMNIST additionally in average precision), Palma and Gini perform way worse than anomaly score and both normality scores. Overall, as the number of anomalous examples grows, the performance of normality measures decreases, performance of anomaly measure increases slightly, and performance of Palma and Gini increases by a large margin.

For diverse inlier settings, Palma and Gini coefficients outperform almost everything for all cases except CIFAR10 0.1% and 1%, KuzijishiMNIST 0.1% in which Gini and Palma coefficients respectively perform the second best to $N_{pp}$ (Table 3 and Supplementary Table 2). As in the diverse outlier settings, the proposition of the previous study^12^ that plain capsule network performs poorly for anomaly detection stands. As in the diverse outlier case, Palma and Gini coefficients are very close to each other.

**Table 3:** AUROCs for diverse inlier setup (fractions 0.1%, 1% and 10%).

	0.1% mean ± std	1% mean ± std	10% mean ± std	Dataset
Palma	**0.6985** ± 0.0570	0.7980 ± 0.0624	**0.9068** ± 0.0413	CIFAR10
Gini	0.6982 ± 0.0575	**0.7984** ± 0.0622	**0.9084** ± 0.0413	
Plain	0.5000 ± 0.0000	0.5131 ± 0.0157	0.7060 ± 0.0768	
A	0.5111 ± 0.1465	0.5217 ± 0.1388	0.6329 ± 0.1361	
$N_{pp}$	**0.7013** ± 0.0465	**0.8157** ± 0.0587	0.8341 ± 0.0284	
$N_{re}$	0.5113 ± 0.1419	0.5223 ± 0.1347	0.6397 ± 0.1250	
ABC	0.5230 ± 0.118	0.5335 ± 0.1163	0.5195 ± 0.1051	
NL	0.5000 ± 0.0812	0.5000 ± 0.0812	0.5000 ± 0.0811	
Palma	**0.9849** ± 0.0080	**0.9981** ± 0.0043	0.9697 ± 0.0035	MNIST
Gini	**0.9848** ± 0.0081	**0.9981** ± 0.0012	**0.9997** ± 0.0002	
Plain	0.8039 ± 0.0727	0.9634 ± 0.0138	0.9928 ± 0.0031	
A	0.9508 ± 0.0342	0.9898 ± 0.0080	0.9819 ± 0.0442	
$N_{pp}$	0.9496 ± 0.0568	0.6210 ± 0.1288	0.3073 ± 0.0997	
$N_{re}$	0.9593 ± 0.0248	0.9920 ± 0.0052	**0.9939** ± 0.0111	
ABC	0.5420 ± 0.2011	0.5398 ± 0.2025	0.5598 ± 0.1960	
NL	0.5048 ± 0.1731	0.5028 ± 0.1722	0.5061 ± 0.1723	
Palma	**0.9067** ± 0.0282	**0.9771** ± 0.0125	**0.9925** ± 0.0053	KMNIST
Gini	0.9066 ± 0.0285	**0.9771** ± 0.0125	**0.9934** ± 0.0056	
Plain	0.6029 ± 0.0322	0.8303 ± 0.0288	0.9558 ± 0.0159	
A	0.7611 ± 0.0875	0.8908 ± 0.0389	0.9501 ± 0.0294	
$N_{pp}$	**0.9248** ± 0.0268	0.8451 ± 0.0402	0.5218 ± 0.0594	
$N_{re}$	0.8147 ± 0.0670	0.9286 ± 0.0242	0.9784 ± 0.0119	
ABC	0.5000 ± 0.1279	0.5000 ± 0.1279	0.5000 ± 0.1279	
NL	0.5000 ± 0.1071	0.5000 ± 0.1074	0.5000 ± 0.1077	
Palma	**0.9289** ± 0.0424	**0.9617** ± 0.0265	**0.9480** ± 0.0582	FMNIST
Gini	**0.9284** ± 0.0426	**0.9661** ± 0.0296	**0.9891** ± 0.0136	
Plain	0.6517 ± 0.1410	0.8076 ± 0.1266	0.9376 ± 0.0525	
A	0.7474 ± 0.1705	0.8192 ± 0.1257	0.8527 ± 0.0923	
$N_{pp}$	0.8762 ± 0.1262	0.7358 ± 0.2231	0.5970 ± 0.2339	
$N_{re}$	0.7374 ± 0.2045	0.7989 ± 0.1598	0.8427 ± 0.1199	
ABC	0.5515 ± 0.2192	0.5499 ± 0.2133	0.5775 ± 0.2011	
NL	0.5000 ± 0.2215	0.5000 ± 0.2208	0.5000 ± 0.2214	

The best and the second-best results are in [bold].

### Malignant skin lesion classification

This constitutes the first application of supervised anomaly detection with capsule networks to a real-world non-benchmark dataset. Following footsteps of Quinn et al.^3^, we consider that anomaly detection can facilitate the search for actual biological anomalies—malignant skin lesions. The main conceptual difference from this work^3^ (apart from using photos instead of transcriptomics data) is that we actually use the examples of such anomalies—the detection is not unsupervised.

The results, as Table 4 shows, are rather similar to the results on the MNIST-like benchmarks (Tables 2 and 3). Palma and Gini outperform every other metric by a large margin and provide almost the same performance. For the case B, diverse outliers and homogeneous inliers, Gini outperforms Palma, but not very much. The $N_{pp}$ measure performs close to Palma and Gini, while the rest is far behind.

**Table 4:** AUROCs for HAM10000 with the following setups: A—diverse outliers, diverse inliers, B—diverse outliers, homogeneous inliers, C—homogeneous outliers, homogeneous inliers, D—homogeneous outliers, diverse inliers.

	A	B	C	D
Palma	**0.7348** ± 0.0160	**0.7312** ± 0.0137	**0.7880** ± 0.0165	**0.7532** ± 0.0209
Gini	**0.7347** ± 0.0157	**0.7312** ± 0.0137	**0.7883** ± 0.0163	**0.7528** ± 0.0207
Plain	0.5000 ± 0.0000	0.5000 ± 0.0000	0.4996 ± 0.0005	0.5000 ± 0.0000
A	0.5693 ± 0.0120	0.5815 ± 0.0216	0.5846 ± 0.0129	0.5953 ± 0.0247
$N_{pp}$	0.6950 ± 0.0055	0.7204 ± 0.0120	0.7468 ± 0.0099	0.7406 ± 0.0152
$N_{re}$	0.5675 ± 0.0122	0.5945 ± 0.0200	0.5848 ± 0.0132	0.6079 ± 0.0234
ABC	0.5456 ± 0.0027	0.5888 ± 0.0300	0.5710 ± 0.0076	0.6066 ± 0.0016
NL	0.5865 ± 0.0030	0.5908 ± 0.0046	0.6173 ± 0.0037	0.6269 ± 0.005

The best and the second-best results are in [bold].

Analysis of average precision (Supplementary Table 3) for this task also show clear superiority of Gini and Palma over the other metrics, closely mirroring the AUROC results, but the difference here is also more pronounced, because every metric except for Gini and Palma scores only about 2% more on average than the respective proportion of the positive class (anomaly). Such result is close to the one we would expect from a degenerate model that outputs 1 regardless of the input.

### CRISPR-Cas9 off-target detection

For the CRISPR off-target task we get the best results with Palma ($0.9631\, \pm \, 0.0125$ AUROC, $0.6876\, \pm \, 0.0264$ average precision) and Gini ($0.9666\, \pm \, 0.0118$ AUROC, $0.6571\, \pm \, 0.0318$ average precision) coefficients and the worst results with ABC and NL ($0.5314 \, \pm \, 0.0134$, $0.5147 \, \pm \, 0.0483$ AUROC respectively, and whopping $0.0271\, \pm \, 0.0007$, $0.0264 \, \pm \, 0.0044$ average precision respectively). Normalized reconstruction error $N_{re}$ again performs worse than its $N_{pp}$ pair—$0.6756 \, \pm \, 0.0372$ AUROC, $0.2725 \pm 0.0328$ average precision, and $0.9147 \, \pm \, 0.0144$ AUROC, $0.304 \, \pm \, 0.0701$ average precision respectively, but anomaly score and plain capsules give not the worst, but the average quality in AUROC—$0.6131 \, \pm \, 0.0471$ and $0.7518 \, \pm \, 0.0404$ respectively, while in average precision, plain capsules score below Gini and Palma only ($0.4059\, \pm \, 0.034$). Anomaly score performs in average precision similarly to AUROC—slightly worse than $N_{re}$ ($0.2535 \, \pm \, 0.0327)$. The advantage of inequality-based measures over the rest is clearly seen.

## Discussion

This paper is based on the parallels between economic inequality and the inequality in the coupling coefficients of capsule networks with dynamic routing by agreement. We apply income inequality analysis to the couplings and propose two metrics for capsule network-based anomaly detection.

Inequality in the size of coupling coefficients arises naturally in Capsule Networks. If we consider Capsule Networks an ensemble of weak learners, the secondary capsules are not supposed to treat all primary capsules equally in considering their signal as an evidence for presence of an object of a particular class in the image, so they have to weight them to achieve specialization.

Another way to look at routing in capsule networks is through the Hebbian learning lenses. Routing, especially routing-by-agreement, is conceptually similar to the idea of Hebbian learning^25^: “neurons that fire together, wire together”. In case of Capsule Networks, connections between primary and secondary capsules that give large scalar product between weighted output vectors grow larger with the training time. Such behavior also leads to larger connections between well learned class capsule and its primary counterparts, and to smaller connections otherwise.

This behavior is closely related to “polarization problem”^13^: tendency of Capsule Networks to converge to a single route from primary to class capsules during training. While it could be a problem for usual classification, polarization seems beneficial for anomaly detection—we observe lack of polarization as the inequality in couplings, we can catch it, measure and use as an anomaly detection tool.

Our inequality-based approach extends the previously proposed metrics based on differences in class probability. It shows an advantage over the competing methods, especially on real-world datasets we considered. We have tested our approach on MNIST-like benchmarks and two complex real-world tasks: skin malignant lesion detection and CRISPR off-target cleavage identification. The experiments show that the proposed methods perform significantly better than other Capsule Networks-based anomaly detection metrics and baselines in most cases. For rather simple datasets (MNIST-like except CIFAR10), in the case of high imbalance (0.1% outliers), the economic inequality methods perform below the anomaly and normality measures though, but it doesn’t happen with more complicated datasets. We hypothesize that there are two possible reasons for such pronounced superiority of Palma and Gini coefficients. First of all, information averaged away by other methods seems actually helpful for solving imbalanced classification. Secondly, there is an inherent inequality within the computations of the network formed by the statistical differences between the abundance of rare and common classes, and this inequality gets exploited by Gini and Palma coefficients which can be seen as ways to indicate the class rareness.

An obvious direction for future studies would be replication of the current study with models based on different routing mechanism (OptimCaps routing^26^, EM routing^27^, spectral capsules^28^, attention-based routing^29^ and others). We predict that the results would be close since the inequality in connection strength is a necessary outcome of a Hebbian-like learning approach (and it is typical behavior in ensemble learning). Another direction is the further exploration of inequality’s nature and the invention of other metrics based on various aspects of it.

## Supplementary Information

Supplementary Information.

## Supplementary Information

The online version contains supplementary material available at 10.1038/s41598-022-17734-7. Source code for the reproduction of the paper is available at https://github.com/bakirillov/capsule_ad.
