# Supplementary material for: Measuring internal inequality in capsule networks for supervised anomaly detection
Source: Sci Rep. 2022 Aug 9;12:13575. doi: 10.1038/s41598-022-17734-7 (PMC9363486; doi:10.1038/s41598-022-17734-7)
Supplement: Supplementary file 1 — Supplementary Information. [file 41598_2022_17734_MOESM1_ESM.pdf]

Measuring Internal Inequality in Capsule Networks for Supervised Anomaly Detection

Bogdan Kirillov[1,2]

Maxim Panov[3,1]

[1]Skolkovo Institute of Science and Technology, Moscow, Russia
[2]Center for Precision Genome Editing and Genetic Technologies for Biomedicine, Institute of Gene Biology, Russian Academy of Sciences, Moscow 119334, Russia
[3]Technology Innovation Institute, Abu Dhabi, United Arab Emirates

Supplementary Note

Weights are initialized at random and are adjusted via Gradient Descent (we use Adam[?] algorithm with basic Pytorch settings). We take the loss parameter $\alpha = 0.0005$ in all the experiments. For HAM10000 study, we resize the images from 900x450 to 100x75 due to hardware constraints. We have not used any kind of data augmentation to train the networks, but we did a number of changes in the original architecture to be able to train the models on HAM10000 images and off-target data. The architectures differ the most in the preprocessing module. We use the same m^+, m^- and λ as the work of Sabour et al.[?] – $0.9, 0.2$ and 0.5. For CIFAR10 and HAM10000 we use batch size of 64 due to hardware constraints, for everything else we use 256. For MNIST-like datasets, the number of primary capsules is 1152, for CIFAR10 is 2048, for PENG is 352, for HAM10000 is 40320. We train CRISPR off-target models for 50 epochs, and we train the rest for 10 epochs. We use early stopping with patience of 3 everywhere. We use ELU instead of ReLU in HAM10000 due to better convergence. For MNIST-like datasets, we have a single input channel, for HAM10000 we have 3 input channels and for CRISPR off-targets we have 4 input channels. Reconstruction subnetwork is a two-layer feed-forward network with ReLU activation. The one we use for MNIST-like trials has 512 and 1024 units in the hidden layers. The network for HAM10000 task has 512 and 1024 units as well. The network for OT task has 128 and 256 units. For CRISPR off-targets we have set the kernel size for preprocessing convolutions and for primary capsules to (2,2). Size of reconstruction for CRISPR off-targets is 184, for MNIST-like is 784, for CIFAR10 is 3072, for HAM10000 is 22500. For both HAM10000 and off-target studies we train 10 networks on different random 90% subsets of the training set to get the estimation of performance metric standard deviation. For MNIST-like datasets including CIFAR10, we estimate the mean and standard deviation of performance metric (AUC) by averaging over values for a model trained to use each class as inlier/outlier. For ABC, we use the same architecture the original ABC study[?] did – a multilayer perceptron with two hidden layers of 300 and 100 neurons with tanh activations for encoder, same perceptron, but with 100 and 300 neurons, tanh activation, for decoder, the output of decoder is sigmoid. We also use the same training regime – 300 epochs with early stopping. For NL, we use the same architecture as Munawar et al.[?] – a pair of Restricted Boltzmann Machines with hidden (for encoder, and visible for decoder) layer of size 500 and Sigmoid activation for the hidden layer. We train this architecture with Contrastive Divergence.

Supplementary Table 1

Table 1. Average precision (area under Precision-Recall curve) for outlier setup (fractions 0.1%, 1% and 10%)

	CIFAR	MNIST	FMNIST	KMNIST	Proportion
Palma	0.9398 ± 0.0144	0.9794 ± 0.0121	0.9795 ± 0.0165	0.9637 ± 0.0095	0.1
Gini	**0.94 ± 0.0144**	0.9791 ± 0.0123	0.98 ± 0.0163	0.9634 ± 0.0097	0.1
Plain	0.9 ± 0.0	0.9157 ± 0.0118	0.9152 ± 0.0234	0.901 ± 0.002	0.1
A	0.9227 ± 0.0247	**0.9898 ± 0.0072**	**0.9857 ± 0.0087**	0.9606 ± 0.0151	0.1
N_{pp}	**0.9408 ± 0.017**	0.9896 ± 0.0081	0.9855 ± 0.0074	**0.9777 ± 0.0079**	0.1
N_{re}	0.9227 ± 0.0243	**0.9948 ± 0.0032**	**0.9881 ± 0.0117**	**0.979 ± 0.0094**	0.1
ABC	0.9382 ± 0.0164	0.9724 ± 0.0163	0.9615 ± 0.035	0.9252 ± 0.0266	0.1
NL	0.9009 ± 0.0272	0.9022 ± 0.0456	0.903 ± 0.0577	0.9011 ± 0.0322	0.1
Palma	**0.9573 ± 0.013**	**0.9978 ± 0.0013**	**0.9931 ± 0.0055**	**0.9889 ± 0.0043**	1.0
Gini	**0.9573 ± 0.013**	**0.998 ± 0.0014**	**0.9931 ± 0.0055**	0.9887 ± 0.0043	1.0
Plain	0.9001 ± 0.0002	0.9728 ± 0.0116	0.9605 ± 0.0159	0.9328 ± 0.0138	1.0
A	0.923 ± 0.0267	0.9939 ± 0.0043	0.9884 ± 0.0084	0.966 ± 0.0102	1.0
N_{pp}	**0.9598 ± 0.0128**	0.9942 ± 0.0037	0.9855 ± 0.0103	**0.9907 ± 0.0036**	1.0
N_{re}	0.9231 ± 0.0274	0.9976 ± 0.0017	0.9887 ± 0.0124	0.9838 ± 0.0077	1.0
ABC	0.9381 ± 0.0188	0.9725 ± 0.016	0.9616 ± 0.0347	0.9256 ± 0.0267	1.0
NL	0.9008 ± 0.0272	0.9027 ± 0.0455	0.903 ± 0.0578	0.9011 ± 0.0318	1.0
Palma	**0.9766 ± 0.0073**	0.9978 ± 0.0024	**0.9955 ± 0.0041**	**0.9958 ± 0.0031**	10.0
Gini	**0.9768 ± 0.0073**	**0.9998 ± 0.0001**	**0.9975 ± 0.0032**	**0.9967 ± 0.0024**	10.0
Plain	0.9273 ± 0.0127	0.9964 ± 0.0012	0.9841 ± 0.0111	0.9811 ± 0.0073	10.0
A	0.933 ± 0.0189	0.9943 ± 0.0049	0.987 ± 0.0143	0.967 ± 0.0122	10.0
N_{pp}	0.9681 ± 0.0092	0.962 ± 0.0137	0.9586 ± 0.0164	0.9643 ± 0.0108	10.0
N_{re}	0.9342 ± 0.0187	**0.9987 ± 0.0011**	0.9856 ± 0.0176	0.9861 ± 0.0061	10.0
ABC	0.94 ± 0.0167	0.9744 ± 0.0163	0.9618 ± 0.0353	0.9284 ± 0.0287	10.0
NL	0.9009 ± 0.0272	0.9023 ± 0.0454	0.903 ± 0.0576	0.9012 ± 0.0322	10.0

Supplementary Table 2

Table 2. Average precision (area under Precision-Recall curve) for inlier setup (fractions 0.1%, 1% and 10%)

	CIFAR	MNIST	FMNIST	KMNIST	Proportion
Palma	**0.2626 ± 0.075**	**0.9678 ± 0.0163**	**0.7999 ± 0.1351**	0.7909 ± 0.0559	0.1
Gini	**0.2631 ± 0.0753**	**0.9677 ± 0.0164**	**0.8008 ± 0.137**	**0.7912 ± 0.056**	0.1
Plain	0.1 ± 0.0	0.6468 ± 0.1244	0.3726 ± 0.2409	0.2852 ± 0.0549	0.1
A	0.1055 ± 0.0385	0.8299 ± 0.0774	0.387 ± 0.281	0.3974 ± 0.1148	0.1
N_{pp}	0.2608 ± 0.0741	0.9121 ± 0.0699	0.7423 ± 0.1469	**0.8206 ± 0.0431**	0.1
N_{re}	0.1082 ± 0.0429	0.8577 ± 0.0611	0.394 ± 0.2972	0.4746 ± 0.091	0.1
ABC	0.1237 ± 0.0641	0.1434 ± 0.1153	0.1421 ± 0.0786	0.1128 ± 0.0503	0.1
NL	0.1076 ± 0.0308	0.1214 ± 0.0737	0.1269 ± 0.0634	0.1101 ± 0.0419	0.1
Palma	0.4201 ± 0.1159	**0.9854 ± 0.02**	**0.8763 ± 0.0916**	**0.9425 ± 0.0205**	1.0
Gini	**0.4224 ± 0.1151**	**0.9944 ± 0.0028**	**0.8898 ± 0.1006**	**0.9427 ± 0.0203**	1.0
Plain	0.1227 ± 0.0274	0.933 ± 0.0246	0.6483 ± 0.2308	0.692 ± 0.0512	1.0
A	0.1368 ± 0.0658	0.9675 ± 0.0176	0.4849 ± 0.2694	0.6855 ± 0.1169	1.0
N_{pp}	**0.4419 ± 0.1131**	0.4527 ± 0.1078	0.5839 ± 0.191	0.7059 ± 0.0544	1.0
N_{re}	0.1373 ± 0.066	0.973 ± 0.0134	0.495 ± 0.2928	0.7975 ± 0.047	1.0
ABC	0.1298 ± 0.0744	0.1441 ± 0.1197	0.1392 ± 0.0752	0.1128 ± 0.0503	1.0
NL	0.1078 ± 0.0313	0.1196 ± 0.0699	0.1268 ± 0.0632	0.1105 ± 0.0428	1.0
Palma	**0.6534 ± 0.133**	0.923 ± 0.0599	**0.851 ± 0.1007**	**0.9763 ± 0.0116**	10.0
Gini	**0.6615 ± 0.1325**	**0.9986 ± 0.001**	**0.9421 ± 0.0702**	**0.98 ± 0.0128**	10.0
Plain	0.4024 ± 0.1258	**0.9809 ± 0.0066**	0.8347 ± 0.1378	0.9023 ± 0.0284	10.0
A	0.2424 ± 0.1311	0.9358 ± 0.1257	0.485 ± 0.2583	0.7676 ± 0.1253	10.0
N_{pp}	0.408 ± 0.0582	0.116 ± 0.0225	0.2688 ± 0.13	0.2549 ± 0.0583	10.0
N_{re}	0.2464 ± 0.1273	0.9647 ± 0.0555	0.5183 ± 0.2766	0.912 ± 0.0395	10.0
ABC	0.1183 ± 0.0575	0.1524 ± 0.1254	0.1471 ± 0.079	0.1128 ± 0.0503	10.0
NL	0.1077 ± 0.0309	0.1223 ± 0.0747	0.1267 ± 0.0632	0.1102 ± 0.0422	10.0

Supplementary Table 3

Table 3. Average precision (area under Precision-Recall curve) for HAM10000 with the following setups: A – diverse outliers, diverse inliers, B – diverse outliers, homogeneous inliers, C – homogeneous outliers, homogeneous inliers, D – homogeneous outliers, diverse inliers.

	A	B	C	D
Palma	**0.3389 ± 0.0329**	**0.2029 ± 0.0132**	**0.4806 ± 0.0493**	**0.2647 ± 0.0319**
Gini	**0.3382 ± 0.0312**	**0.2032 ± 0.0131**	**0.4812 ± 0.0485**	**0.2637 ± 0.0319**
Plain	0.1893 ± 0.0	0.1095 ± 0.0	0.2165 ± 0.0	0.1378 ± 0.0
A	0.2251 ± 0.0042	0.1541 ± 0.0051	0.2631 ± 0.0039	0.1892 ± 0.0072
N_{pp}	0.2694 ± 0.0067	0.1912 ± 0.0142	0.3545 ± 0.0132	0.2357 ± 0.0202
N_{re}	0.2268 ± 0.0049	0.1598 ± 0.0037	0.266 ± 0.0045	0.1977 ± 0.0064
ABC	0.2081 ± 0.0008	0.1438 ± 0.0009	0.2579 ± 0.0032	0.1945 ± 0.0006
NL	0.2254 ± 0.0014	0.1388 ± 0.0017	0.2834 ± 0.0023	0.1972 ± 0.0026
Proportion	0.20072	0.118202	0.200584	0.118061

References

[1] Kingma, D. P. & Ba, J. Adam: A method for stochastic optimization. *arXiv preprint arXiv:1412.6980* (2014).

[2] Sabour, S., Frosst, N. & Hinton, G. E. Dynamic routing between capsules. In *Advances in neural information processing systems*, 3856–3866 (2017).

[3] Yamanaka, Y., Iwata, T., Takahashi, H., Yamada, M. & Kanai, S. Autoencoding binary classifiers for supervised anomaly detection. In *Pacific Rim International Conference on Artificial Intelligence*, 647–659 (Springer, 2019).

[4] Munawar, A., Vinayavekhin, P. & De Magistris, G. Limiting the reconstruction capability of generative neural network using negative learning. In *IEEE 27th International Workshop on Machine Learning for Signal Processing*, 1–6 (2017).
